# Dual-task effects of walking-speed on inhibitory control and decision-making under risk

**DOI:** 10.1038/s41598-025-88497-0

**Published:** 2025-04-22

**Authors:** Carlotta Maiocchi, Marta Milanesi, Nicola Canessa, Stefania Sozzi, Giulia Mattavelli, Antonio Nardone, Claudia Gianelli

**Affiliations:** 1 IUSS Cognitive Neuroscience (ICON) Center, Scuola Universitaria Superiore IUSS Pavia, Pavia, Italy; 2 Istituti Clinici Scientifici Maugeri IRCCS, CognitiveNeuroscienceLaboratoryofPaviaInstitute, Pavia, Italy; 3 Department of Computer, Electrical and Biomedical Engineering, University of Pavia, Pavia, Italy; 4 Centro Studi Attività Motorie and Neurorehabilitation and Spinal Units of Pavia Institute, Istituti Clinici Scientifici Maugeri IRCCS, 27100 Pavia, Italy; 5 Department of Clinical-Surgical, Diagnostic and Pediatric Sciences, University of Pavia, Pavia, Italy; 6 Department of Clinical and Experimental Medicine, University of Messina, Messina, Italy

**Keywords:** Cognitive-motor-interference, Dual-task, Decision-making, Inhibitory control, Walking speed, Human behaviour, Decision, Cognitive control, Motor control

## Abstract

The effect of simultaneously performing two tasks (dual-task effects, DTEs) has been extensively studied, mainly focusing on the combination of cognitive and motor tasks. Given their potentially detrimental impact on real-life activities, the impact of DTEs has been investigated in both healthy individuals and patients. In this Registered Report, we aimed to replicate previous DTEs when a task requiring executive-inhibitory skills is involved while also expanding the evidence on basic facets of decision-making. We recruited 50 healthy young participants who performed a stop-signal task and two gambling tasks (loss-aversion and risk-aversion) while sitting and while walking at three treadmill speeds (normal, slow and fast). We report a significant difference in performance during single-task and dual-task, although with high individual variability. The data show no effect of the walking speed on all the cognitive tasks. Analyses on postural alignments, assessed in the cadence, gait cycle length and stance phase, confirm previous results on cognitive prioritization strategies of healthy individuals. Based on our results, we highlight the need to further investigate prioritization strategies when tasks involving higher cognitive functions are performed along a motor task in healthy individuals and patients with the aim of offering targeted training and rehabilitation protocols.

The stage 1 protocol for this Registered Report was accepted in principle on 28/06/22. The protocol, as accepted by the journal, can be found at: 10.17605/OSF.IO/5MWH7.

## Introduction

The ability to successfully perform two tasks simultaneously, e.g., walking while talking or texting, is a staple of many everyday activities. Performing two concomitant activities entails, however, resources exceeding the mere sum of those required by the two tasks in isolation. The resulting dual-task effects (DTEs) can either reflect a significant improvement or deterioration in the dual-task condition compared to a single-task one, i.e., dual-task costs (DTCs) or benefits (DTBs), respectively^1^. Most of the available evidence on DTEs results from paradigms combining a cognitive and a motor task, leading to the definition of DTCs as cognitive-motor interference (CMI). It is noteworthy that even gait parameters such as speed and cadence are influenced by a concurrent cognitive effort^2,3^, suggesting that—rather than being completely automatic—walking requires central control^4^.

Given their possible detrimental impact on real-life activities, DTEs have received increasing attention both in healthy participants and neurological patients—e.g., in chronic stroke^5^, Parkinson’s disease^6,7^, and other neurodegenerative conditions^8^—and were tested in a wide range of tasks, from simple lab- based conditions (e.g., counting while standing) to more ecological ones (e.g., texting while walking). Notably, while DTEs have been reported in both healthy and pathological populations, the magnitude of these effects—and consequently their potential impact on daily and working activities—appears larger in older adults and patients. In this sense, the extent of DTEs has been linked to a decay in the flexibility of attentional allocation and thus in task prioritization based on the complexity of the required motor behaviour^6^. Two competing models, i.e., “serial bottleneck” and “capacity sharing”, appear more likely to explain the emergence of DTEs and predict their direction (e.g., in terms of DTCs). The former posits that only one information process can be active at a time, and thus that two or multiple tasks cannot effectively take place in parallel^9^. As to the latter, tasks can be rather managed in parallel, but control capacity must be shared across tasks. The resources allocated to each task would be thus reduced compared to single-task conditions, and in most dual-task instances—because of task requirements or other contextual and personal factors—this leads to prioritizing one over the other^10^.

Several facets of DTEs and CMI remain, however, largely unexplored. First, most studies have so far focused on motor function as the primary outcome measure, with more limited, and more variable^6^, reports of their effects on cognitive functioning^1,11^. Unveiling the impact of DTEs in the cognitive domain and on the combination of cognitive-motor tasks as a whole^12^—would however be crucial to determine the short- and long-term effects of training protocols and interventions, particularly in neurological patients^13^. Second, the effect of performing motor tasks on cognitive functioning has been mainly investigated or interpreted in terms of executive skills. For instance, it has been shown that slowed overground walking is accompanied by decreased performance, whereas fast motorized treadmill walking is characterized by improved performance. In this sense, it has been claimed that opposite requirements are at play in terms of effortful executive-attentional control^4^ in these two exemplary cases.

However, it is still largely unknown whether, and to what extent, CMI also affects other facets of higher-order cognitive functioning. In this sense, existing evidence points to decision-making under risk as an exemplary—yet still largely unexplored—benchmark for DTEs. In fact, increased motor cautiousness is expected to reduce risk-taking behaviour because of shared executive control of the two processes, particularly when inhibitory motor control is involved^14^. By combining decision-making with a simple motor control task, Verbruggen and colleagues^14^ showed that not only challenging motor tasks—such as those inducing a more cautious behaviour—reduced gambling, but also that a short inhibitory training reduced risk taking for a few hours afterwards. Notably, while the impact of various factors on decision-making—including the concurrent performance of a demanding cognitive task—has been extensively investigated, the evidence on motor tasks is far more limited^15^. As to dual-task conditions, there is evidence that cognitive tasks with a high executive load have a detrimental impact on decision behaviour^16,17^. When motor demands are at play, a balancing task produces more disadvantageous performances compared to sitting^15^, as a result of increased attentional and control requirements even in the absence of any prioritization instruction. Overall, while promising, the available evidence in this domain is still limited, and several factors should be considered when addressing decision-making in a dual-task framework.

First, at least some facets of judgment and valuation processes have been shown to rely on individual differences in executive skills^18^, and the depletion of executive resources appears to enhance spontaneous decision-making biases^19^ such as the preference for avoiding losses over acquiring gains (i.e., loss aversion^20^). This evidence therefore suggests that the previously reported CMI effects on executive functioning might extend to decision-making. Second, the latter is inherently intertwined with action selection^21^, and valuation processes have been shown to shape kinematic features of the choice-related motor response, such as hand reaching or mouse cursor trajectories^22–26^. Previous proposals about the reverse effect, i.e., a modulation of decision-making by motor constraints on action selection^27,28^, have been only partially supported by the evidence that perceptual decision-making is biased towards the options associated with the lowest motor costs^29,30^. Whether concurrent motor performance also affects reward-based decision-making is largely unknown, and to the best of our knowledge the only study addressing this issue reported no significant effect of motor costs on intertemporal preferences in a delay-discounting paradigm^31^.

On these grounds, the present study aimed to investigate the interplay between motor performance and basic decision-making processes such as anticipating and weighing negative against positive consequences, as well as certain against probabilistic outcomes. The former trade-off, typically associated with variable degrees of loss aversion^20^, is also influenced by individual differences in the typical preference for certain compared with probabilistic outcomes (i.e., risk aversion^32^). We assessed the extent and direction of DTEs associated with walking on a motorized treadmill (at normal, slowed, or increased speed) while concurrently performing a gambling-task tracking the degree of loss- aversion). In addition to the gambling-task, we also employed a stop-signal task to extend the evidence on the role of executive-inhibitory skills in DTEs, and to directly test the role of individual differences in capacity sharing (task prioritization) across different tasks.

Specifically, we investigated three main experimental questions and tested our predictions within a Bayesian analytical framework (see Table 1 for a detailed Design outline).

**Table 1:** Design table.

Question	Hypothesis (if applicable)	Sampling plan (e.g., power analysis)	Analysis plan	Interpretation given to different outcomes
(1) Does the combination of treadmill-walking and different types of cognitive tasks (stop-signal and gambling tasks) elicit DTEs compared to their execution in isolation?	HP1: For each of the outcome variables, we expect to elicit DTEs with a significant change in performance regardless of other experimental manipulations (i.e., all treadmill- walking speed conditions)	The sampling plan is based on a bayesian sequential design with maximal N. Data collection will continue until a pre-determined level of evidence is obtained (BF > 10), or a maximal number of participants is reached (50 participants = 200 sessions)	Two one-sample bayesian t-tests, one for each of the outcome variables, on the absolute values of DTE (%) tested against a value = 0	(1) BF_10_ > 10 for all tasks → Moderate or strong evidence of DTEs in all cognitive tasks. This finding replicates the existing literature on the stop-signal task and confirms that DTEs extend to gambling (2) BF_10_ > 10 only for the stop- signal task → Moderate or strong evidence of DTEs for this specific task, replicating the existing literature, but insufficient evidence of DTEs in gambling. If concomitantly BF_01_ > 10 for the decision- making variable, then moderate to strong evidence in favour of H_0_ → gambling tasks are not affected by a concurrent motor task, possibly due to the fact that gambling tasks engage attentional and inhibitory processes other than those involved in motor control (3) BF_10_ > 10 only for the decision-making tasks → moderate to strong evidence that DTEs could be elicited by the decision-making tasks. If concomitantly, BF_01_ > 10 for the stop-signal test → moderate to strong evidence that for this task we were unable to elicit any DTEs failing to replicate the existing literature (4) BF_10_ < 10 for either tasks → the evidence is inconclusive on that specific task or both (5) BF_01_ > 10 for both tasks. Moderate to strong evidence that our task combinations did not elicit any DTEs, possibly due to the type of motor task (treadmill walking) that might be less sensitive to DTEs compared to overground walking or balance tasks
Does treadmill walking at different speeds affect participant’s performance in a stop-signal task?	HP2a: Treadmill walking at a faster-than-normal speed will improve participants’ behavioural performance, thus decreasing SSRTs (positive DTE (%) values). On the contrary, treadmill walking at slower than normal speed will interfere with the cognitive task, increasing SSRTs (negative DTE (%) values)	The sampling plan is based on a bayesian sequential design with maximal N. Monitoring of BFs will be performed on the three tests used for HP1	(1). One paired- sample bayesian, one tail, t-test comparing DTE (%) calculated for the stop-signal SSRTs between normal and fast speed. 2. One paired- sample bayesian one tail, t-test comparing DTE (%)calculated for the stop-signal SSRTs between normal and slow speed	(1). BF_10_ > 10 for test (1) → Moderate to strong evidence that treadmill walking at a faster-than-normal speed improves participant’s inhibitory performance 2) BF_10_ > 10 for test (2) → Moderate to strong evidence that treadmill walking at a slower speed interferes with participant’s inhibitory performance. (3). BF_10_ < 10 for test (1) → The evidence on this manipulation is inconclusive. (4) BF_10_ < 10 for test (2) → The evidence on this manipulation is inconclusive. (5) BF_01_ > 10 for test (1) → Moderate to strong evidence that treadmill walking at a faster-than-normal speed does not affect participant’s inhibitory performance in the stop-signal task. (6) BF_01_ > 10 for test (2) → Moderate to strong evidence that treadmill walking at a slower-than-normal speed does not affect participant’s performance in the stop-signal task
Does treadmill walking at different speeds affect participant’s decision making in a gambling task?	HP2b: Treadmill walking at a faster-than-normal speed significantly enhances participants’ gambling, with reduced loss aversion (negative DTE (%) values). On the contrary, treadmill walking at a slower-than-normal speed produces decreased gambling and thus increases loss aversion (positive DTE (%) values)	The sampling plan is based on a bayesian sequential design with maximal N. Monitoring of BFs will be performed on the three tests used for HP1	Two paired-sample bayesian, one-tail, t-tests comparing DTE (%) calculated for loss aversion (λ) between (1) normal and fast speed, (2) normal and slow speed	(1) BF_10_ > 10 in test (1) → Moderate to strong evidence that walking at a faster-than-normal speed significantly impacts decision- making, with increased gambling, possibly due to a shift of executive resources from the motor to the cognitive task.BF_10_ < 10 for (1) → The evidence for this test is inconclusive. 2) BF_10_ > 10 in test (2) → Moderate to strong evidence that walking at a slower-than-normal speed significantly impacts decision making (reduced gambling) possibly due to increased demand for executive control under this condition. (4) BF_10_ < 0 in test (2) → The evidence is inconclusive. (5) BF_01_ > 10 in test (1) → Moderate to strong evidence that walking at a faster-than-normal speed does not impact gambling. (6) BF_01_ > 10 in test. (2) → Moderate to strong evidence that walking at a slower-than-normal speed does not impact gambling

1. Does the combination of treadmill-walking and different types of cognitive tasks (stop-signal and gambling tasks) elicit DTEs (in %) compared to their execution in isolation?

HP1: For each of the outcome variables, we expected to elicit DTEs with a significant change in performance regardless of other experimental manipulations (i.e., all treadmill-walking conditions). Specifically, we predicted significant DTEs for both tasks, thus replicating the existing literature (stop-signal task) and extending it to the decision- making domain (loss aversion).

2. Does treadmill walking at different speeds affect participant’s performance in a stop-signal and in a gambling task?

HP2a: Treadmill walking at a faster-than-normal speed would improve participants’ behavioural performance, thus decreasing the stop-signal reaction time (SSRT) compared to normal speed (positive DTE% values). On the contrary, we predicted that treadmill walking at a slower-than-normal speed would interfere with the cognitive task, increasing SSRTs compared to walking at a normal speed (negative DTE% values). These predictions were in line with previous evidence that increasing/decreasing gait speed with respect to one’s own spontaneous pace is associated with improved/worsened executive performance^4^. In addition, it has been already shown that reduced walking speed increases the attentional demand due to the reduced gait automaticity and higher cortical demand with changes in muscular activation patterns^33^. Another factor potentially interfering with cognitive performance is represented by the increased demands on postural stability during slow walking speed^34^. On the contrary, we expected that walking at a faster speed would improve participants’ performance because of diminished attentional demands^33^ leading to prioritizing the cognitive task.

HP2b: based on the evidence that the depletion of executive resources facilitates spontaneous biases in decision-making^19^, and stopping motor responses decreases gambling in multitask situations^35,36^, we predicted that treadmill walking at a slower-than-normal speed would produce reduced gambling, i.e., increased loss aversion (positive DTE % values). On the contrary, treadmill walking at a faster-than-normal speed was expected to increase executive control over the spontaneous avoidance of risk, thus prioritizing a challenging motor task, and thus to result in decreased loss aversion (negative DTE % values).

## Methods

### Design

In a within-subjects design, participants underwent four experimental sessions in which we assessed single and dual-task performance in a series of cognitive tasks combined with a motor one (i.e., walking on a treadmill at different speeds). A first, single-task session, was followed by three others where the different walking speeds and cognitive tasks were matched in a completely randomized design. For technical reasons, data collection was not performed blind to the experimental conditions.

#### Loss-aversion

To assess the modulation of decision-making valuation processes by walking at different speeds, participants were asked to perform a gambling task involving the evaluation of real prospective monetary gains and losses^37^. In each trial, they were asked to choose between a risky mixed-gamble (resulting in equally probable, i.e., p = 50%, outcomes) and a guaranteed alternative (i.e., a certain outcome). To estimate loss aversion, a task included 49 gain–loss trials requiring choosing between a gamble with equally probable variable gains or losses and a guaranteed alternative of 0^38^. The possible outcomes were sampled from a 7 × 7 matrix with uncorrelated gains and losses, centred on each participant’s “baseline” indifference point (detected in the first session; see Experimental procedure).

Risk-aversion was estimated through 30 gain-only trials requiring choosing between a guaranteed gain and a gamble with equally probable positive and 0 possible outcomes. Since we aimed to prevent the effect of behavioural learning from past outcomes, and thus focus on “decision utility”^39–41^, gambles were not resolved immediately. Namely, subjects were informed that a) missed response would result in the automatic acceptance of the gamble; b) all the accepted gambles would be covertly played by the computer, and c) the outcome of one randomly extracted trial from each task would determine the final monetary payoff (see Experimental procedure). In both tasks, gambles were shown for 3000 ms and separated by an intertrial interval ranging between 500 and 1500 ms (average: 1000). The expected duration was thus 196 and 120 s for the loss- and risk-aversion tasks, respectively.

#### Stop-signal task

The modulation of executive control skills by walking at different speeds was measured using a visual version of the stop-signal task (SST^42,43^). This task drives participants to provide a “go” response which must be occasionally inhibited when the “go” stimulus is followed, after a variable stop-signal-delay (SSD), by a “stop” signal. Dynamically adjusting the delay between go and stop signals allows modulating task difficulty. As suggested by current best practices to ensure the elicitation of a prepotent motor response^44,45^, the task design entailed low inhibition probability, fast trial pace and individual SSD tracking.

Each trial began with a fixation cross lasting 500 ms, followed by a green left- or right-ward arrow (go stimulus). Participants were instructed to respond as fast and accurately as possible to the arrow direction by pressing, with their index or middle finger, the buttons of a handheld response device (see Experimental procedure). On 25% of trials, the go stimulus was followed—at a variable SSD—by a stop-signal, here represented by the arrow turning from green to red. Participants were instructed that responding correctly and quickly, and stopping successfully, were equally important. Regardless of the arrow direction, the SSD was initially set to 200 ms, and dynamically adjusted in 50-ms increments to achieve a p (stop|signal) ≈ 0.5. This procedure indeed entailed that the SSD was increased and decreased after successful and failed stops, respectively. Participants performed 3 blocks of 60 trials (thus 135 go and 45 stops overall), each lasting 3,000 ms. The task duration was thus 540 s.

#### Motor task

The motor task consisted in treadmill walking at different speeds. The preferred walking speed was individually assessed (in the first session; see Experimental procedure) and then used to determine faster- than-normal and slower-than-normal walking speed parameters to be used in the subsequent sessions. For the purpose of subsequent exploratory analyses—not included in the pre-registration—motor behaviour in this experiment was assessed through an accelerometer, an inertial sensor that measures acceleration along its sensitive axis^46^.

Kinematic data were recorded using the BTS G-WALK (BTS Bioengineering S.p.A., Garbagnate Milanese, Italy), a portable, wireless, inertial system with a wearable sensor. The device is composed of a triaxial accelerometer (16 bit/axes) with a sensitivity level of ± 2 g, a triaxial gyroscope (16 bit/axes) with a sensitivity level of ± 2000◦/s, a triaxial magnetometer (13 bit, ± 1,200 μT), and a global positioning system receiver. Data were recorded at 100 Hz frequency. This wireless inertial sensor, housed in a specialized belt, was placed on the participant’s lower back with the center of the device positioned at the first sacral vertebrae (S1). The participant was completely free to walk at different speeds, while spatiotemporal data were transmitted by Bluetooth to a computer, and subsequently processed using the BTS G-Studio software. At the end of each session, an automatic report containing the gait parameters recorded during the test was displayed. The mean and standard deviation of the following measures was evaluated: cadence (steps/min), gait cycle time (s), gait cycle length (m), stance phase (% gait cycle), swing phase (% gait cycle), single stance phase (% gait cycle, for left and right foot), single swing phase (% gait cycle, for left and right foot).

#### Quality check

To control for the proper implementation of the procedure, and to assure compliance with the instructions, for each session and across the various blocks/tasks we randomly presented an additional 10% (with respect to the total N of task-specific trials) of control trials. In these trials, we asked participants to solve simple arithmetic operations (single-digit additions and subtractions) by choosing between two possible results presented in the left- or right-bottom corner of the screen. Responses were given by pressing either the left or the right button of the same handheld device used for all the other tasks. For each participant/session, the averaged accuracy for these trials served as: (1) procedure quality checks (e.g., ensuring proper functioning of stimuli presentation/response devices and correct recording of the responses), and (2) positive controls to assure participant’s responsiveness and compliance with the instructions.

### Experimental procedure

All participants took part in four experimental sessions: the first one focused on single-task measurements, and the following three on dual-task ones. As to the cognitive tasks, stimuli were presented on a 17” LCD screen placed in front of the participants. Answers were recorded by means of a handheld response device, allowing participants to respond using the index and middle finger. Stimuli presentation and response recording were managed by the software Eprime v3.0 (Psychology Software Tools, Pittsburgh, PA).

#### Single-task session

In the first session participants were asked to perform the stop-signal, gambling, and motor tasks separately.

As to the motor task, we first determined their preferred walking speed. They were asked to walk on a treadmill while an operator increased the rate of speed of 0.2 m/s every 5 s in a stepwise manner up to the preferred walking speed (i.e., the one at which participants feel comfortable walking ≈ 3,2 km/h ± 0,50). To ensure that the appropriate normal walking speed has been reached, participants were then asked to rest for 5 min, then to walk again on the treadmill and confirm that they would be comfortable walking for 15 min at that speed. This self-selected speed value was taken as a reference to determine a faster walking pace (preferred walking speed increased by 20%) and a slower one (preferred walking speed decreased by 20%). Finally, participants were asked to walk on the treadmill for 2 min at each of the three walking speeds to ensure their suitability. These individually determined speed parameters were used in all the following three dual-task sessions. For safety purposes, participants always wore a harness while walking on the treadmill.

As to cognitive tasks and control trials, participants were informed that their performance would determine the final monetary payoff by increasing (up to double) or decreasing (up to erase) an initial task-specific endowment. In the single-task session the stimuli for the loss aversion task were initially centered to an indifference ratio of 2, which, notwithstanding considerable individual differences, is widely acknowledged as a reliable average estimate at the population level^41,47–49^. The stimuli for subsequent sessions were then individually tailored to the thereby computed participant’s subjective indifference point.

#### Dual-task session

Across the three dual-task sessions, each cognitive task was matched with a different treadmill-walking speed (normal, low, high) and presented in separate blocks. The resulting blocks were presented in randomized order (simple randomization with each combination presented once) across sessions for each participant. As data collection depended upon sequential analyses, no counterbalancing across participants was possible.

To avoid a possible effect of fatigue, each session was followed by a 3-day pause before the following one. In the same vein, each session was designed to allocate an adequate number of breaks between blocks.

### Sampling plan

The sampling for this study was based on a Bayesian sequential design with maximal N^50^. In this design, data collection continued until (1) a pre-determined level of evidence (Bayes Factor) was obtained, or (2) a maximal number of participants was reached. We opted for this design as an efficient alternative to open-ended sequential designs, given the high number of planned sessions (four per participant) that might limit resources availability (e.g., reducing the pool of potentially available participants while increasing the risk of dropouts compared to single-session experiments).

The threshold for evidence was set at Bayes Factor > 10, while the maximal N was determined a priori using a Bayes Factor Design Analysis with Fixed N (https://tellmi.psy.lmu.de/felix/BFDA_app) by implementing the following parameters to obtain a profile of Distribution of Default Bayes Factors.

- Bayes Factor > 10
- Expected effect size = 0.8
- Sample size: 50
- Decision Boundaries:
  - Lower Boundary: 0.1
  - Upper Boundary: 10
- Prior on Effect Size:
  - Default Prior: Cauchy (μ = 0, r = p2/2)

The expected effect size was determined based on the converging evidence from the existing literature and prior research from our group^4^, employing similar manipulations and reporting large effect sizes. Based on the obtained distribution, we estimated that if the expected effect size is 0.8 and the default prior on effect size is used for analyses, we would need at least 50 observations to obtain a Bayes factor larger than 10 with a probability of p = 0.9. As this would correspond to 200 sessions in total, we deemed this estimation suitable both for our experimental design and the expected resource allocation for this study. Bayes factors were updated in a sequential fashion after each completed participant until the threshold of BF > 10 in all monitored tests or the maximal N were reached, whichever came first.

Since our statistical analyses involved several tests, we monitored only those connected to HP1 (see Table 1). Namely, we monitored those tests—one for each of the outcome variables—aimed at determining whether our design was capable of inducing DTEs in all the cognitive tasks/measures, regardless of the additional experimental manipulations.

Following this sampling plan, we recruited healthy young adults (aged 18–40 years). Participants with a current or past diagnosis of neurological and/or motor diseases that might affect the correct execution of the tasks were excluded from the recruitment. Similarly, we excluded participants practicing sport activity at a competitive or professional level, as this might affect the homogeneity of the sample. Only participants completing all four sessions were included in the analyses. Participants failing to comply with the motor task or withdrawing their consent, during or after completion of all sessions, were discarded. Since positive control trials were designed to assess participants’ attentiveness and compliance with instructions, only participants with a mean accuracy > 90% were retained for analyses. Exclusion criteria for the remaining participants were based on their performance in the cognitive tasks (see below).

### Analysis plan

Data analyses were performed blind to the experimental conditions using custom-made scripts.

#### Loss aversion

Participants’ choices in the gambling tasks were assessed under the assumption that both gain-loss and gain-only trials are required to isolate loss aversion from risk attitude, since both are involved in anticipatory valuation processes^37^. Therefore, both gain-loss and gain-only trials associated with a same walking speed were simultaneously fitted to the following Prospect-theory-inspired model^20^ through maximum likelihood estimation as implemented in MATLAB (MathWorks, Natick, MA):

1 $$u(x^{ + } ) = p \times (x^{ + } )^{\rho }$$

2 $$u(x^{ - } ) = - \lambda \times p \times ( - x^{ - } )^{\rho }$$

3 $$p(gamble) = \frac{1}{{1 + e^{ - \mu } \times \left( {u(gamble) - u(guaranteed} \right)}}$$

The subjective utilities associated with gains (x +) and losses (x-) are computed with Eqs. (1) and (2) (p = 0.5 in the present study) and expressed through a parameter ρ embodying risk attitude in terms of the diminishing marginal sensitivity to value with probabilistic outcomes. Loss aversion is quantified by lambda (λ), the multiplicative weight associated with anticipated losses compared with gains. The thereby computed utilities of the gamble and certain alternative outcome are then used to estimate risk attitude (ρ) and loss aversion (λ) via maximum likelihood estimation, with the Eq. (3). Since λ is positively skewed, log(λ) is considered in subsequent analyses of loss aversion. Loss-seeker participants with λ < 1, and those for whom the maximum likelihood procedure did not converge after 50,000 iterations for any of the estimated parameters (suggestive of a lack of fit for the model and thus representing a possible indicator of inconsistent choices), were discarded from further analyses.

#### Stop-signal task

Following previous studies^44^, performance was assessed in terms of a) go-trial reaction time (GoRT); b) failed stop-trial reaction time (FsRT); c) mean rate of stopping, i.e., p(stop|signal); and d) stop-signal reaction time (SSRT).

The first three values were used for a quality-check, based on the predictions of the race model^43^ that a) GoRT should be longer than FsRT; b) due to the SSD staircase method, individual p(stop) should approximate 0.5. On these grounds, we excluded participants for whom GoRT are faster than FsRT, or p(stop|signal) falls outside the 0.4—0.6 range. As previously suggested^35^, SSRT was then calculated (a) after excluding trials with RTs higher than 2,000 ms (missing responses); (b) based on the block-wise integration method, i.e. SSRT = (nth RT − mean SSD), which entails estimating SSRT for each block separately and then compute the average of these four estimates. This approach has been shown to provide more reliable estimates of the latency of response inhibition compared both with the “mean” method (in which SSRT = mean RT − mean SSD), and with the “experiment-wise” calculation (i.e., SSRT computed over all blocks), because it is more resistant to the bias introduced by the skewed distribution of reaction times and the gradual slowing of response latencies^35^.

#### Calculation of dual-task effects

Dual-task effects were calculated separately for each cognitive task, outcome variable (loss aversion λ, and SSRT) and walking condition (normal, low, fast speed) using the formula DTE (%) = [(dual-task − single-task)/single-task]*100. For the SSRT, the formula was adjusted^1^ to account for the fact that higher SSRT indicates worse performance as follows: DTE (%) = [− (dual-task − single-task)/single-task] *100.

For SSRT, by convention, negative DTE values indicated a dual-task cost (interference) and positive values indicated a dual-task benefit (facilitation). As to decision-making parameters, positive and negative DTE (%) values reflected increased and decreased loss aversion, respectively.

### Statistical analyses

Statistical analyses were performed on DTE (%) using Bayesian t-tests (see Table 1). Bayes Factors with default priors were calculated to quantify the observed evidence in terms of odds ratio between the null and the alternative hypothesis evaluated against a threshold of BF ≥ 10 (below = anecdotal evidence, above = moderate to strong (if > 30) evidence. We conventionally indicated the evidence in favour of the alternative hypothesis BF_10_ and the evidence in favour of the null hypothesis BF_01_.

As to HP1, we performed three one-sample Bayesian t-tests, one for each of the outcome variables (SSRT, ρ and λ) on the absolute values of DTE (%) against a test value = 0.

As to HP2a, we used two paired-sample Bayesian, one tail, t-tests comparing DTE (%) calculated for the stop-signal task SSRTs between (1) normal and fast speed, (2) normal and slow speed.

As to HP2b, we performed two paired-sample Bayesian, one-tail, t-tests comparing DTE (%) calculated for loss aversion (λ) between (1) normal and fast speed, (2) normal and slow speed.

## Results

Data were collected between November 2022 and April 2023. Results are presented as mean ± standard deviation (SD). All statistical tests were performed using the software JASP (V. 0.17.2.0, Jasp Team 2023), R (V. 4.3.0) and Matlab (V. 2020). A total of 50 healthy young participants were recruited (demographics are reported in Supplementary Table S1). Four participants failing the quality check (i.e., accuracy rates < 90% on all arithmetic tests across the three tasks) were excluded from any further analyses. For the SST, 16 participants with a *presp* [p(stop|signal)] that fell outside the range of 0.4–0.6 in all conditions were excluded from the main analyses. A total of 30 participants (19 females, 11 males) were therefore included in the final analyses of the SST data. For the LA and RA tasks, we excluded 4 participants because of either (a) lack of convergence of maximum likelihood estimation after 50,000 iterations for any of the parameters, (b) λ <1 (loss-seeking), or (c) λ >10 (suggestive of a tendency to reject all gambles)^51^. For each of the assessed variables, we checked the assumptions of an approximately normal distribution, and four participants were also excluded because of DTE values with a kurtosis that exceeded 5 standard deviations of the mean. A total of 38 participants were therefore included in the final analyses of decision-making data.

### Effects of treadmill walking on cognitive performance (HP1)

For each of the outcome variables, we expected to elicit DTEs with a significant change in performance regardless of other experimental manipulations (i.e., all treadmill-walking speed conditions).

**Fig. 1:**
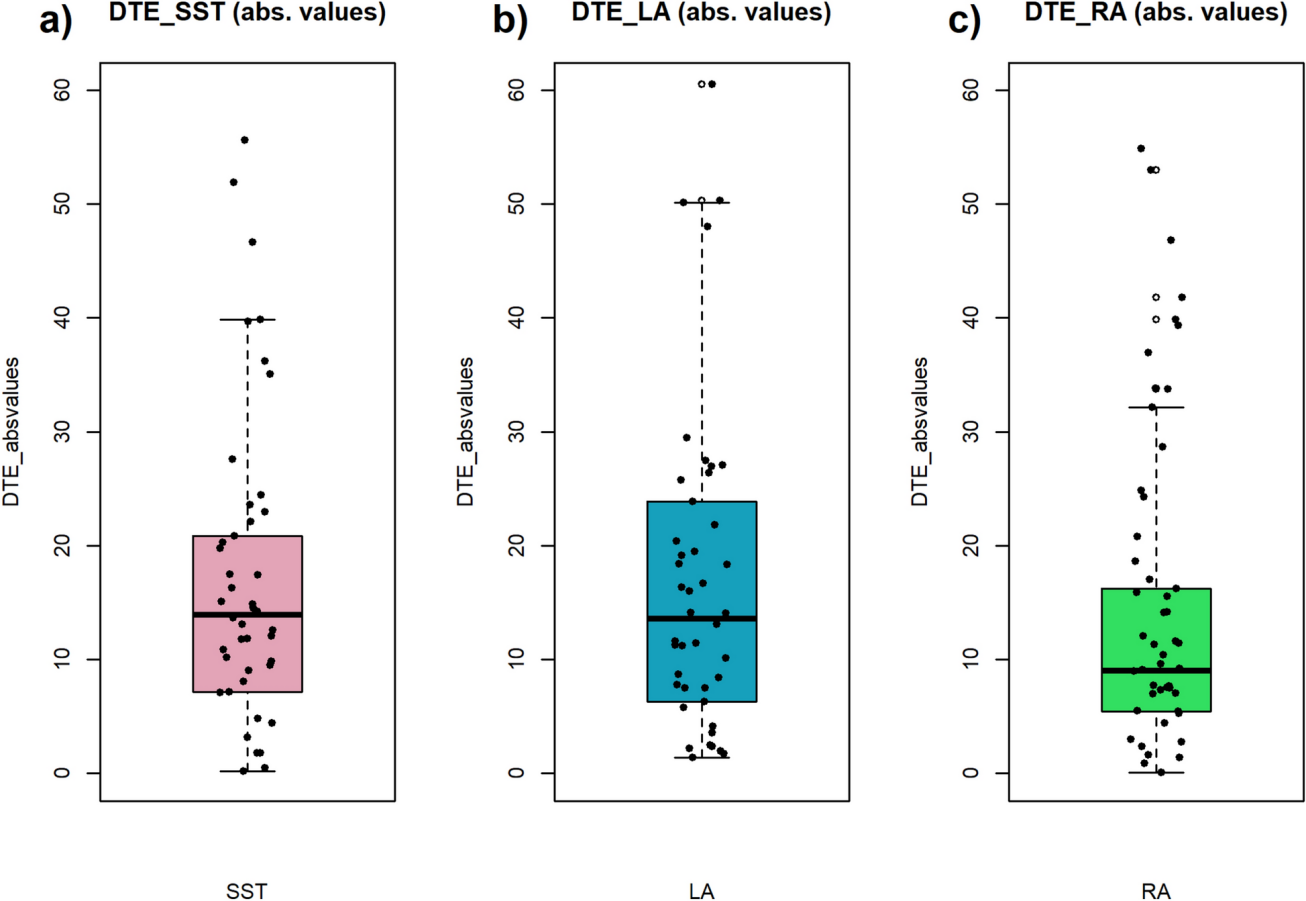
Absolute values of DTE (%) in SST, LA and RA cognitive tasks. In the first panel (**a**) the DTE (%) against the test value = 0 is represented; (**b**) represents DTE (%) in LA and (**c**) shows RA.

The directional one-sample Bayesian t-test on the absolute values of DTE (%) tested against a value = 0 showed strong evidence in support of our first hypothesis for both SST and decision-making tasks. This evidence replicates the existing literature on executive functions (SSRTs BF_10_ = 663000) and extends it to loss aversion (λ BF_10_ = 1.007x10^6^) and risk aversion (ρ BF_10_ = 171406; see Table 2 and Fig. 1).

**Table 2:** Descriptive statistics for HP1.

	N	BF_10_	Error %
DTE_SSRT (%)	30	6,662,865	1.150 × 10^–10^
DTE_λ (%)	38	1.007 × 10^6^	8.522 × 10^–12^
DTE_ρ (%)	38	171,406	8.355 × 10^–11^

### Effects of different treadmill speeds on executive functioning (HP2a)

**Fig. 2:**
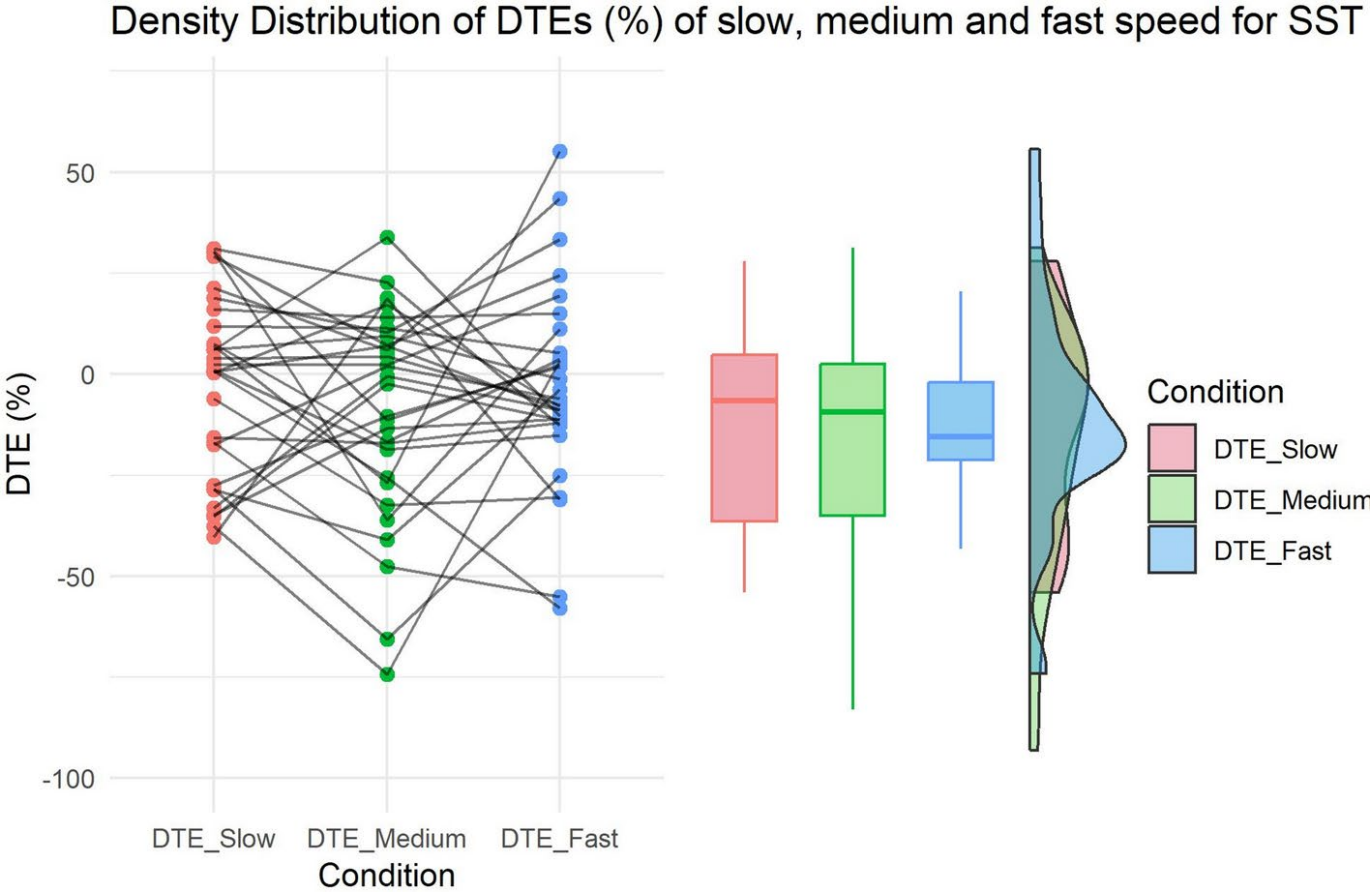
HP2a—Effect of Treadmill speed on executive functions. Raincloud plots show the distribution of DTEs (%) as difference between normal and faster-than-normal speed, and between normal and slower-than-normal speed.

Walking at a faster-than-normal speed increased on average the DTE, improving the performance on the SST (average DTE at normal speed:  − 9.093 ± 25.8, average DTE at fast speed: − 3.255 ± 24.39). However, the paired-sample directional t-test revealed a Bayes factor (BF_10_) of 0.521, thus not providing any evidence in favour of our hypothesis (Fig. 2).

Contrary to our expectations, walking at a slower-than-normal speed increased on average the DTE, improving the performance on the SST (DTE at normal speed: − 9.093 ± 25.8, DTE at slow speed: − 3.24 ± 22.43). However, the paired-sample Bayesian directional t-test did not provide any evidence supporting our hypothesis (BF_10_ = 0.097). In contrast, when considering the Bayesian t-test in the opposite direction, a BF_01_ of 10.33 highlighted strong evidence for the null hypothesis (descriptive statistics are reported in Table 3).

**Table 3:** Descriptive statistics for HP2a and HP2b.

SSRT	N	Mean	SD	Coefficient of variation	95% credible interval
DTE_NORMAL (%)	30	− 9.093	25.819	− 2.839	(− 18.734 to 0.548)
DTE_FAST (%)	30	− 3.255	24.397	− 7.496	(− 12.364 to 5.855)
DTE_SLOW (%)	30	− 3.238	22.433	− 6.927	(− 11.615 to 5.138)
λ					
DTE_NORMAL (%)	38	7.725	26.533	3.435	(− 0.996 to 16.446)
DTE_FAST (%)	38	10.366	22.482	2.169	(2.977 to 17.756)
DTE_SLOW (%)	38	9.959	31.738	3.187	(− 0.473 to 20.391)

### Effects of different treadmill speeds on decision-making (HP2b)

**Fig. 3:**
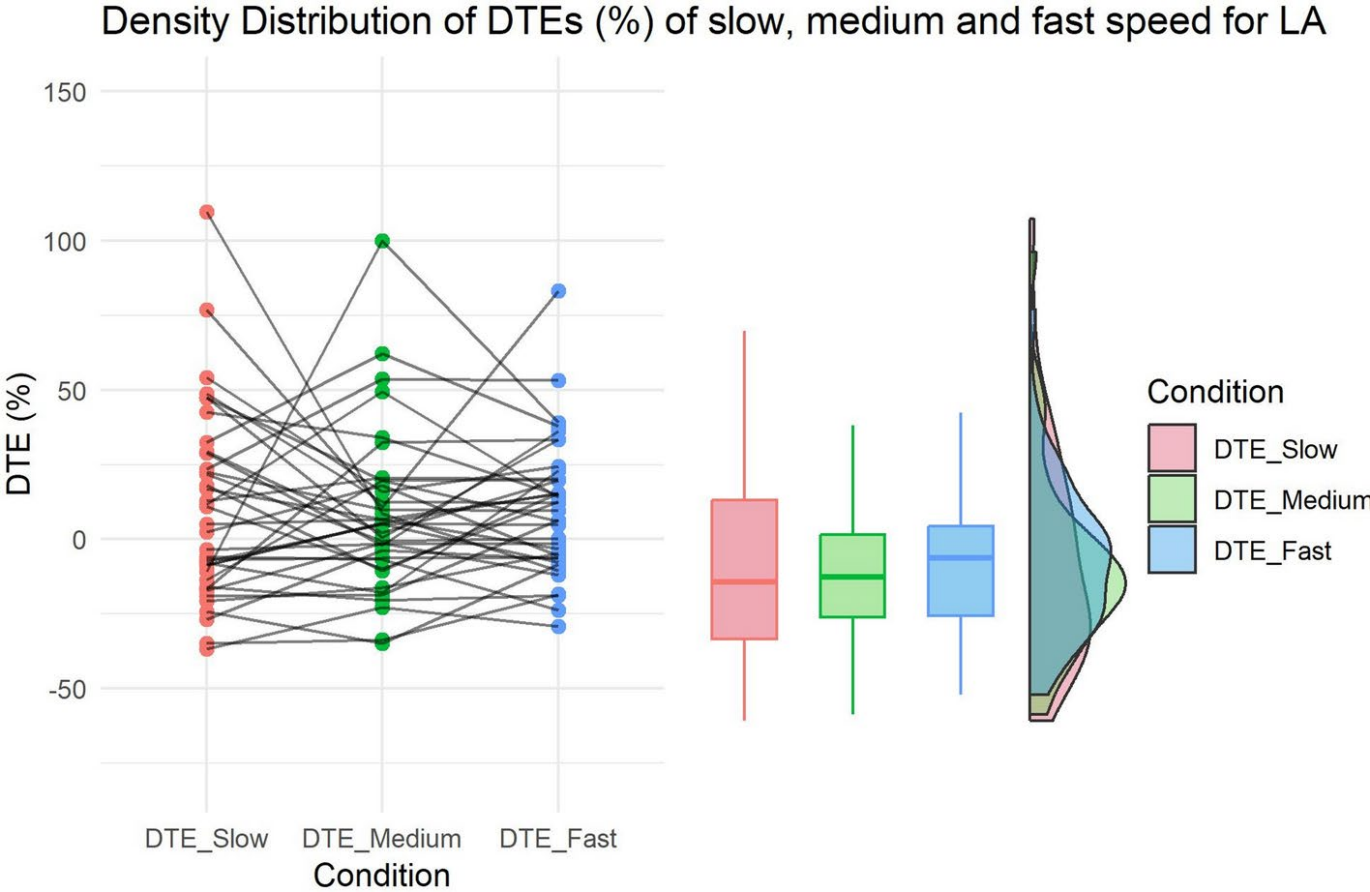
HP2b—Effect of Treadmill speed on decision-making (LA). Raincloud plots show the distribution of DTEs (%) as difference between normal and faster-than-normal speed, and between normal and slower-than-normal speed.

The paired-sample Bayesian directional t-test comparing DTE (%) at normal and faster speed provided no evidence for our hypothesis (BF10 = 0.111) (Fig. 3): contrary to our predictions, loss aversion was found to increase at a faster-than-normal speed (average DTE at normal speed = 7.725, average DTE at fast speed = 10.366). A Bayesian t-test in the opposite direction indeed provided substantial evidence in favour of the null hypothesis (BF01 = 9.04).

Similarly, a paired-sample Bayesian directional t-test provided no evidence for our hypothesis also regarding the comparison between normal and slower-than-normal speed (BF10 = 0.243). Notably, a general increase of loss aversion was found during slow– as compared to normal–speed (DTE at normal speed 7.725, DTE at slow speed = 9.959; see descriptive statistics in Table 3).

#### Exploratory analyses

Additional exploratory analyses were carried out to further investigate the observed effects. The main outcomes of these analyses are discussed in this section and reported in full in the Supplementary Materials.

First, we used Bayesian paired sample t-tests to assess risk aversion at the three different walking speeds. Our findings generally replicated the evidence obtained for loss aversion, with increased risk aversion during faster- and slower-than-normal, compared to normal, walking speed (average DTE at normal speed = 2.1, average DTE at fast speed = 3.83, average DTE at low speed = 6.71; see Table S2). However, the paired t-tests did not provide evidence for our hypothesis (BF10 normal vs. fast speed = 0.192, BF10 normal vs. slow speed = 0.277; see Table S3). Figure S1 reports DTEs (%) in all cognitive domains in association with the three different speeds.

Motor performance was analyzed using Bayesian t-tests on DTEs (%), to assess the effect of different cognitive tasks on kinematic variables. Moreover, we used repeated-measures ANOVA to investigate possible interactions between cognitive and motor variables. As motor parameters, we considered cadence, the gait cycle length (GCL) and time (GCT), the single stance phase (SSP), the stance (StP) and the swing (SwP) phase, as well as the step length for the right (RF) and left foot (LF).

A directional Bayesian one-sample t-test, assessing absolute DTE values against a test value of 0, showed strong evidence for the presence of DTEs on all motor variables (Cadence BF10 = 4.55 × 109, GCL BF10 = 6.21 × 107, GCT BF10 = 1.47 × 1010, SSP BF10 = 2.36 × 107, StP BF10 = 8.59 × 106, SwP BF10 = 4.38 × 107, RF BF10 = 53,940 and LF BF10 = 33,128; see Table S4).

The effect of each task on spatiotemporal gait parameters was assessed through Bayesian paired t-tests, comparing DTEs (%) calculated for each motor variable during SST, LA and RA tasks across (1) normal and faster-than-normal speed, (2) normal and slower-than-normal speed. Concerning risk aversion, large effects on single stance phase and stance phase were found when comparing the DTE at normal and slow speed [BF10 (SSP) = 7.34, DTE (SSP) at normal speed = − 0.53, DTE (SSP) at slow speed = − 1.69, BF10 (SP) = 7.41, DTE (SP) at normal speed = 0.355, DTE at slow speed (SP) = 1.132]. For all the other motor parameters we found no evidence of a modulation of DTEs during different cognitive tasks.

A two-way (task-by-speed) Bayesian repeated measure ANOVA was performed on all kinematic outcome variables to evaluate possible interactions among cognitive tasks and motor variables. Only the analyses on Cadence, GCL and GCT showed that the data were respectively 27.75, 1.20 × 10^6^ and 11.41 times more likely under the model including Speed as the predictor, compared to the null model (Table S21). Further post-hoc comparisons resulted in posterior odds in favour of the alternative hypothesis only for cadence when considering speed as a factor (i.e. slower- vs. faster- than normal speed, and normal vs. faster-than normal speed), and in GCL considering the task as a factor (i.e., loss aversion vs. stop signal, and risk aversion vs. stop signal). Comparing the other conditions resulted in moderate evidence supporting the null hypothesis (see Table S22).

Since session order might impact participants’ performance across tasks, we ran an additional analysis of variance (ANOVA) on cognitive data to assess its possible modulation of the observed effects. We considered the condition (single task, slower-than-normal speed, normal speed and faster-than-normal speed) as a fixed factor, and the order in which participants performed the session (day I–II–III–IV) as a random factor. No evidence was found for an effect of session order on results (p(SST) = 0.693, p(LA) = 0.549; see Table S23 and S24).

Finally, we used Pearson’s correlation to assess the strength and direction of a possible relationship between concurrent changes of motor and cognitive performance. To this purpose, we first computed the value of stride length (based on speed and cadence) as a representative outcome for the pace domain^52^. We found SSRT to be positively correlated with cadence (p = 0.04) and negatively correlated with both stride length (p(SL) = 0.04) and gait cycle time (p(GCT) = 0.41; see Table S25). This result shows a strong correlation among the spatial, temporal and spatio-temporal modulations of gait variables by the concurrent performance of executive tasks. Such a pattern was not confirmed for Loss Aversion, since only GCL had a significant correlation with this cognitive task (p = 0.014; see Table S26).

## Discussion

The present study investigated whether walking at different speeds interacts with executive functioning and decision-making in terms of cognitive performance and gait stability and variability. Participants performed a Stop-Signal task and two gambling tasks, both as single-tasks and as part of a cognitive-motor dual-task while walking on a treadmill at three individually optimized speeds: normal, faster than normal, and slower than normal. Dual-task effects were calculated to assess whether and how the different attentional demands and cognitive control associated with these conditions affected cognitive and/or motor performance.

Our first hypothesis (HP1) concerned the presence of DTEs for all cognitive outcome variables (SSRT, λ and ρ) regardless of the other experimental manipulation (treadmill walking speed). The results support our hypothesis that a change in performance occurs when performing motor and cognitive tasks simultaneously rather than separately. While aligning with prior evidence^2,53^, our findings extend the implications of dual-tasking to basic decision-making trade-offs such as those inherent in loss- and risk-aversion. According to the two competing models that have been suggested to underpin DTEs, these results are in line with the hypothesis of serial processing of task performance^54^. Specifically, even if the primary (cognitive) and the secondary (motor) tasks involve different effectors, combining them in a dual-task setting has a larger effect compared to their performance in isolation, and affects not only “basic” executive functions such as motor inhibition but also higher-order cognitive processes involved in decision-making.

**Fig. 4:**
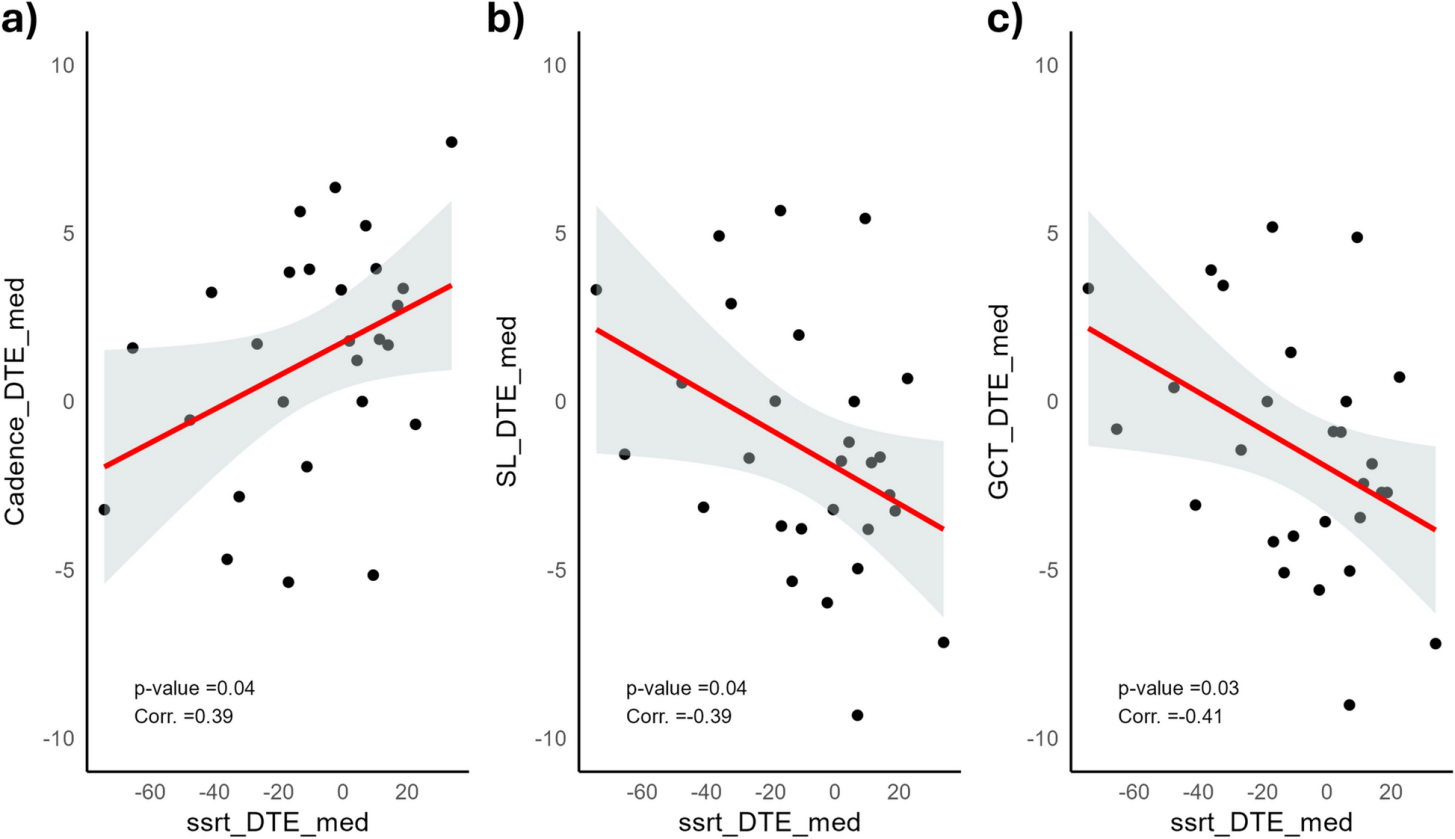
Correlations during the dual-task condition performed in medium speed. The first figure (**a**) shows cadence; (**b**) step length and (**c**) the gait cycle time. Each DTE is correlated with the DTE of the SST during medium speed.

**Fig. 5:**
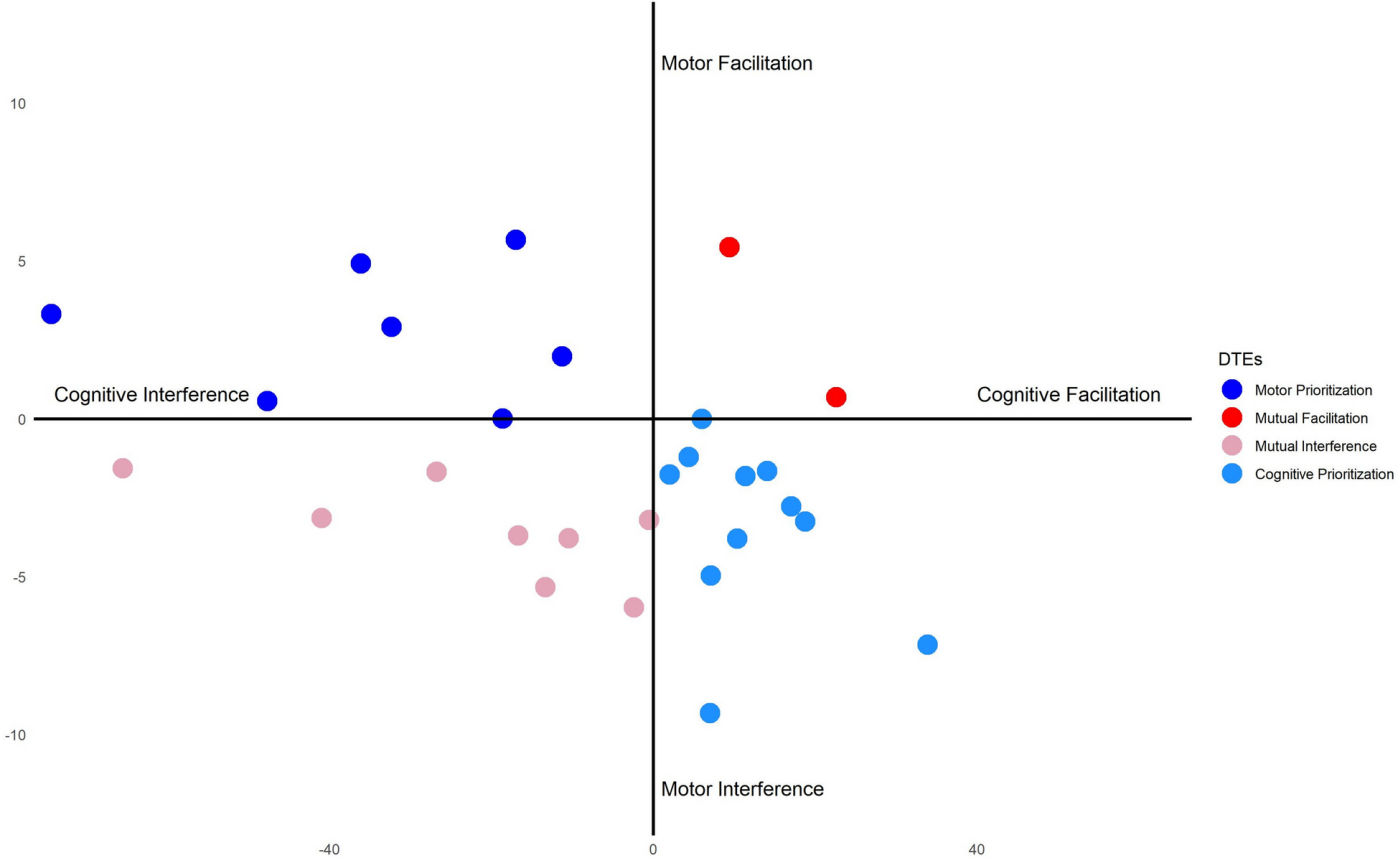
Observed patterns of cognitive-motor interference reported following Plummer & Eskes’ framework. Each measure is expressed as a means of DTE in SST during self-selected speed and stride length.

Our second hypothesis (HP2a and HP2b) concerned a modulation of the direction of DTEs as a consequence of different walking speeds. Namely, we hypothesized that increasing or decreasing walking speed would either increase or decrease cognitive performance. However, our results did not provide any supporting evidence in this regard. Contrary to related studies^33,55^, our findings do not support the hypothesis of a different task prioritization during normal vs. fixed-speed walking. This evidence can be addressed in possible different prioritization strategies that could have impacted on the outcome. It is worth noting that our data highlighted high heterogeneity in cognitive and motor performance across the dual-task conditions (see Fig. 4), with some participants showing a mutual facilitation, or conversely a mutual interference, of cognitive and motor performance, while in a third sub-sample decreased performance for one task is associated with improvement performance in the other. As previously reported^56^, the fact that dual-task manipulations comparably influence motor and cognitive processing within single participants highlights the need to delve into the individual factors shaping individual differences in the direction of DTEs. Individual differences in attentional control, working memory capacity and fluid intelligence have been previously found to modulate arousal and cognitive strategies underlying task performance^57,58^. Accordingly, the present results might have been shaped also by individual differences in the allocation of cognitive resources to the multiple processes inherent in dual-tasking, which provides novels insights into refined DTE models of the interplay between cognitive and motor performance (Fig. 5).

Although we could not report any evidence in favour of our initial HP2a and HP2b, it is worth addressing the general pattern of results that emerged from our study. Specifically, both slow and fast walking speeds were associated with shorter SSRTs, as well as increased loss and risk aversion, compared to the normal speed. This evidence is suggestive of an increased attentional load during speed changes, in turn leading to prioritize the cognitive tasks over the motor one, thus providing further support to recent related evidence^59–62^. Notably, it has been shown that cognitive-motor interference might not occur with low cognitive demands, even in a complex motor condition such as walking. Constant low-intensity motor activity was rather associated with a decreased Stroop effect (i.e., better executive performance) during walking compared to both sitting and standing^60^. Low-intensity activity can thus improve cognitive metrics of selective attention and inhibitory control. In our case, this finding would explain why speed modulations that might in principle interfere with physiological walking did not induce the expected interference in healthy young adults.

Other non-cognitive factors might also have played a role in determining the observed pattern. Arousal, for instance, has been shown to be influenced by the presence of cues promoting motor responses^63,64^. Notably, several studies suggest an association between withholding/cancelling motor responses and the Go/No-Go and Stop Signal tasks^35,64,65^. Theoretical and empirical accounts indeed consider increased arousal to enhance responsiveness, in turn lowering response threshold and favoring action release in the go-and-stop trade-off^66,67^. For instance, it has been shown that motor cautiousness affects impulsive choice tasks, favouring a more cautious decision-making style with risk being assessed as less desirable^66^. The need to exert greater caution on motor responses, and the evaluation of the negative consequences associated with the loss of control, may reduce the value assigned to risk-taking, thus favouring a more risk-averse approach. In line with this interpretation, we reported higher loss-aversion and risk-aversion when participants’ natural walking speed was experimentally manipulated. By requiring higher motor cautiousness and control, this manipulation might increase the degree of interference on cognitive processing of information regardless of its valence^68^.

Overall, our results suggest that decision-making under risk during a concomitant motor performance might reflect in qualitatively different response strategies, as participants appeared to be more risk-averse. Lower attentional demands on postural stability and increased automaticity of gait due to treadmill walking lead to a prioritization of cognitive resources, thereby modulating decision-making strategies towards the usual spontaneous bias against risk-taking^69,70^. In this respect, it is notable that many everyday decision-making situations entail a complex interplay between cognitive and motor processes. Value-based choices are often embedded within complex multitasking environments in which multiple cognitive processes run in parallel with motor ones. The notion of “embodied decision” indeed reflects the possible modulation of decision-making by the current bodily states in terms of physical and motor capabilities^71–73^. As our data are suggestive of increased loss and risk avoidance in combination with motor performance, this evidence highlights the importance of addressing the potential interaction between seemingly distinct processes such as motor and cognitive control.

To investigate further potential interactions between cognitive and motor performance, we performed exploratory analyses to assess DTEs on spatio-temporal gait parameters. Notably, we report strong evidence in favour of the presence of DTEs in all motor variables. In addition, we also showed a strong correlation in the dual-task condition between executive functions and cadence, gait cycle time, as well as stride length. Such a correlation between cognitive performance and gait parameters fits with the existing evidence of significant variations of spatiotemporal gait parameters in healthy young participants when simultaneously performing a cognitive task^55,74^. These modulations are considered to represent a strategy to maintain both optimal movement and cognitive performance and have been reported as a metric of reduced cognitive control during dual-tasking. In neurophysiological terms, this combination of reduced cognitive control on motor performance, and increased cortical activity for cognitive performance, reflects in reduced alpha-frequency activity and increased theta-frequency activity in fronto-central brain regions, suggesting an overall increased cognitive load^62,75,76^.

One possible interpretation of our findings is that participants adopted a “cautious gait” on the treadmill in response to the possible inherent challenges to balance imposed by treadmill walking. Such cautious gait, visible in the modulation in gait cycle, ensured dynamic stability when concurrently performing a challenging cognitive task. Treadmill walking at different speeds likely involves substantial training for participants, and may thus require more cognitive effort (i.e., consciously adjusting one’s position on the treadmill) or greater cortical involvement to control speed, thus likely influencing the amount of attention allocated to the walking task. Decreased walking speed and increased stride time variability have been reported during dual-task walking, and often interpreted as measures of gait stability^77^. As our study involved healthy young adults, these modulations of spatio-temporal variables might reflect participants’ flexible adaptations of their task strategies to optimize cognitive performance.

Importantly, however, treadmill walking is considered less ecologically valid compared with overground walking, due to extensive evidence of mixed results on gait patterns and joint kinematics^78^. Unlike overground walking, which involves variable terrain and dynamic balance adjustments, treadmill walking enforces consistent motor patterns that may reduce attentional demands and stabilize gait. Prior research indeed highlighted differences between treadmill and overground walking, such as increased stance times and altered double-support phases. Due to its relatively automatic, constrained and undisturbed nature, conventional treadmill walking has been shown to interfere less with ongoing cognitive processing than overground walking^79,80^. During dual-task treadmill walking, the mechanical assistance of the treadmill might indeed act as a control mechanism stabilizing the walking pattern, even when attention is directed away from walking. Inferences about brain-behavior relationships drawn from dual-task treadmill walking may therefore not reflect those observed during typical overground walking^79^. Importantly, however, treadmill walking ensures a constant motor load throughout the experimental procedure, thereby representing a reliable technique to evaluate gait or locomotion. In particular, in this experiment it allowed uninterrupted collection of more gait cycles for biomechanical parameters within limited space, as well as an easier use of safety harness during the performance of the cognitive task.

Further research, including more fine-grained motor measurements, is required to clarify how motor and cognitive resources are allocated in dual-task conditions across different populations (e.g., healthy young adults, older adults, and patients) and experimental settings. Similarly, future studies will necessarily have to consider the role of individual strategies in determining the direction and magnitude of dual-task effects, particularly when dealing with higher-order cognitive tasks—such as decision-making—that are inherently shaped by multiple sources of individual differences (e.g., personality traits). The present findings also highlight the need for a better characterization of the factors shaping individual performance, in terms of fine-grained motor and cognitive measurements (e.g., at the single-trial level), their interplay, and concerning their neurophysiological correlates. Such efforts will pave the way for optimized interventions in training and rehabilitation programs, targeting the specific needs of individuals based on their cognitive and motor profiles.

## Supplementary Information

Supplementary Information.

## Data Availability

This study was pre-registered as Stage 1 Registered Report on 14.07.2022 following in-principle acceptance https://osf.io/dcya6. Materials, raw data, and all code necessary to run and analyse this study were publicly shared https://osf.io/5mwh7/.
