# Supplementary material for: Dual-task effects of walking-speed on inhibitory control and decision-making under risk
Source: Sci Rep. 2025 Apr 22;15:13877. doi: 10.1038/s41598-025-88497-0 (PMC12015226; doi:10.1038/s41598-025-88497-0)
Supplement: Supplementary file 1 — Supplementary Information. [file 41598_2025_88497_MOESM1_ESM.docx]

Dual-task effects of walking-speed on inhibitory

control and decision-making under risk

Carlotta Maiocchi, Marta Milanesi, Nicola Canessa, Stefania Sozzi, Giulia Mattavelli, Antonio Nardone, Claudia Gianelli

# Supplementary Materials

Here are reported the demographics of participants (the full sample used in LA analyses is shown) and full data of the exploratory analyses carried out to evaluate possible modulations of risk aversion when performing the task at three different walking speeds, and to investigate a possible modulation on kinematic parameters during dual-task performance. A further analysis was performed to evaluate the possible role of the order of the session as a covariate on the observed effects.

1. **Demographics**

**Table S1.** Demographics for LA sample

|  | Mean | SD |
| --- | --- | --- |
| Age | 25.08 | 3.71 |
| Height _cm_ | 170.14 | 8.54 |
| Mass _Kg_ | 63.44 | 9.67 |
| Sex (Male/Female) | 13/38 |  |

1. **Risk Aversion (RA) – DESCRIPTIVE STATISTICS**

**Table S2**. Descriptive statistics of Bayesian t-tests comparing ρ across different speeds

| ρ | N | Mean | SD | Coefficient of variation | 95% Credible Interval |
| --- | --- | --- | --- | --- | --- |
| DTE_MEDIUM | 38 | 2.100 | 23.159 | 3.757 | (-5.512 9.712) |
| DTE_FAST | 38 | 3.803 | 20.555 | 5.404 | (-2.953 10.560) |
| DTE_SLOW | 38 | 6.707 | 30.788 | 4.590 | (-3.413 16.827) |

**Table S3**. Descriptive statistics of Bayesian factors for comparing ρ across different speeds

|  | N | BF_10_ | Error % |
| --- | --- | --- | --- |
| DTE_Medium vs Fast | 38 | 0.192 | 0.047 |
| DTE_Medium vs Slow | 38 | 0.277 | 0.041 |

**Figure S1**. Effect of Treadmill speed on inhibitory control and decision-making (DTE (%) in slow, medium and fast speed.


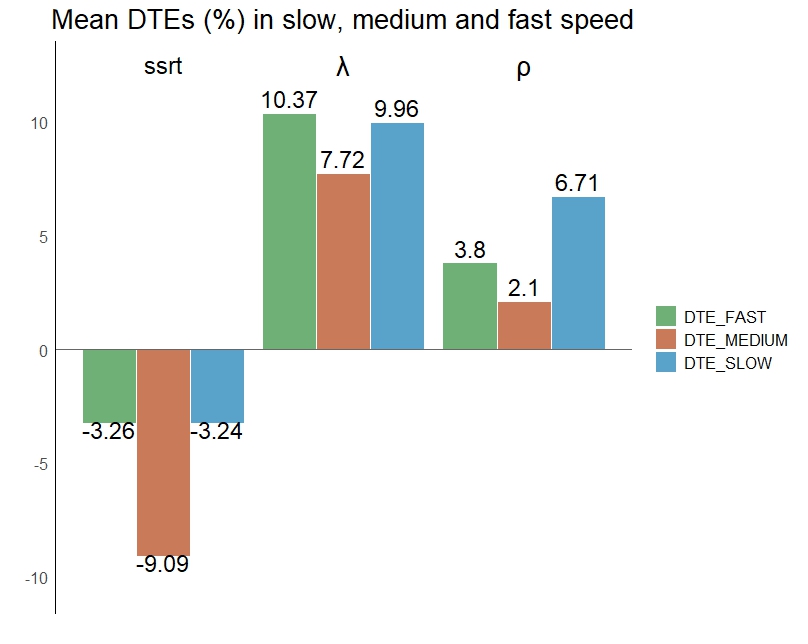


1. **MOTOR ANALYSES**

**One sample t-tests**

A directional Bayesian one-sample t-test was used to assess absolute DTE values against a test value of 0.

**Table S4**. Descriptive statistics of Bayesian factors for comparing ρ across different speeds

|  |  | N | DTE_slow | DTE_Medium | DTE_fast |
| --- | --- | --- | --- | --- | --- |
| Cadence | BF_10_ | 43 | 6.88x10^9^ | 1.97x10^9^ | 8.1x10^7^ |
|  | Error % | 43 | 3.55x10^-14^ | 1.78x10^-12^ | 2.18x10^-13^ |
| GCL | BF_10_ | 43 | 3.65x10^6^ | 4.77x10^7^ | 1.32x10^8^ |
|  | Error % | 43 | 2.76x10^-12^ | 1.91x10^-13^ | 2.17x10^-13^ |
| GCT | BF_10_ | 43 | 8.45x10^7^ | 1.62x10^9^ | 8.95x10^7^ |
|  | Error % | 43 | 2.18x10^-13^ | 1.04x10^-11^ | 2.18x10^-13^ |
| SSP | BF_10_ | 43 | 3.32x10^6^ | 2.26x10^8^ | 66965 |
|  | Error % | 43 | 2.99x10^-12^ | 1.97x10^-13^ | 8.32x10^-11^ |
| StP | BF_10_ | 43 | 1.04x10^6^ | 1.83x10^7^ | 201778 |
|  | Error % | 43 | 1.24x10^-11^ | 1.58x10^-13^ | 3.85x10^-11^ |
| SwP | BF_10_ | 43 | 2.69x10^6^ | 5.83x10^7^ | 70168 |
|  | Error % | 43 | 4.09x10^-12^ | 2.04x10^-13^ | 8.03x10^-11^ |
| RF | BF_10_ | 43 | 518183 | 119815 | 165588 |
|  | Error % | 43 | 2.26x10^-11^ | 5.43x10^-11^ | 4.37x10^-11^ |
| LF | BF_10_ | 43 | 251114 | 101878 | 107392 |
|  | Error % | 43 | 3.37x10^-11^ | 6.09x10^-11^ | 5.86x10^-11^ |

**Paired sample t-tests**

The effect of each task on spatiotemporal gait parameters was assessed through Bayesian paired t-tests, comparing DTEs% calculated for each motor variable during SST, LA and RA across (1) medium and faster than normal speed, (2) medium and slower than normal speed. Here are reported the Bayesian paired samples t-tests and the descriptives for each DTE of motor variables.

**Cadence**

**Table S5**. Bayesian Paired Samples T-Test

| **Measure 1** | **Measure 2** | **BF_10_** | **Error %** |
| --- | --- | --- | --- |
| DTE_LA_med | DTE_LA_slow | 0.179 | 0.055 |
| DTE_LA_med | DTE_LA_fast | 0.513 | 0.034 |
| DTE_RA_med | DTE_RA_slow | 0.239 | 0.049 |
| DTE_RA_med | DTE_RA_fast | 2.329 | 1.101×10^-6^ |
| DTE_SST_med | DTE_SST_slow | 3.154 | 7.677×10^-7^ |
| DTE_SST_med | DTE_SST_fast | 0.387 | 0.039 |

**Table S6.** Descriptive statistics of cadence

|  |  |  |  |  |  | **95% Credible Interval** | |
| --- | --- | --- | --- | --- | --- | --- | --- |
| **Measure** | **N** | **Mean** | **SD** | **SE** | **Coefficient of variation** | **Lower** | **Upper** |
| DTE_LA_med | 43 | 1.074 | 4.551 | 0.694 | 4.237 | -0.327 | 2.475 |
| DTE_LA_slow | 43 | 1.325 | 4.120 | 0.628 | 3.109 | 0.057 | 2.593 |
| DTE_LA_fast | 43 | 0.231 | 2.639 | 0.402 | 11.410 | -0.581 | 1.043 |
| DTE_RA_med | 43 | 1.283 | 4.360 | 0.665 | 3.398 | -0.059 | 2.625 |
| DTE_RA_slow | 43 | 1.676 | 4.918 | 0.750 | 2.935 | 0.162 | 3.189 |
| DTE_RA_fast | 43 | -0.146 | 3.291 | 0.502 | -22.561 | -1.159 | 0.867 |
| DTE_SST_med | 43 | 1.099 | 4.409 | 0.672 | 4.011 | -0.258 | 2.456 |
| DTE_SST_slow | 43 | 2.524 | 5.337 | 0.814 | 2.115 | 0.881 | 4.166 |
| DTE_SST_fast | 43 | 0.283 | 3.578 | 0.546 | 12.636 | -0.818 | 1.384 |

**Gait Cycle Length (GCL)**

**Table S7**. Bayesian Paired Samples T-Test

| **Measure 1** | **Measure 2** | **BF_10_** | **Error %** |
| --- | --- | --- | --- |
| DTE_LA_med | DTE_LA_slow | 0.232 | 0.049 |
| DTE_LA_med | DTE_LA_fast | 0.258 | 0.047 |
| DTE_RA_med | DTE_RA_slow | 0.214 | 0.051 |
| DTE_RA_med | DTE_RA_fast | 0.210 | 0.052 |
| DTE_SST_med | DTE_SST_slow | 1.401 | 0.019 |
| DTE_SST_med | DTE_SST_fast | 0.173 | 0.056 |

**Table S8**. Descriptive statistics of GCL

|  |  |  |  |  |  | **95% Credible Interval** | |
| --- | --- | --- | --- | --- | --- | --- | --- |
|  | **N** | **Mean** | **SD** | **SE** | **Coefficient of Variation** | **Lower** | **Upper** |
| DTE_LA_med | 43 | -1.340 | 6.265 | 0.955 | -4.675 | -3.268 | 0.588 |
| DTE_LA_slow | 43 | -2.394 | 7.866 | 1.200 | -3.285 | -4.815 | 0.027 |
| DTE_LA_fast | 43 | -0.184 | 7.579 | 1.156 | -41.211 | -2.516 | 2.149 |
| DTE_RA_med | 43 | -0.947 | 8.002 | 1.220 | -8.455 | -3.409 | 1.516 |
| DTE_RA_slow | 43 | -1.804 | 8.892 | 1.356 | -4.929 | -4.541 | 0.933 |
| DTE_RA_fast | 43 | 0.089 | 7.473 | 1.140 | 84.144 | -2.211 | 2.389 |
| DTE_SST_med | 43 | -2.130 | 5.291 | 0.807 | -2.483 | -3.758 | -0.502 |
| DTE_SST_slow | 43 | -3.816 | 6.975 | 1.064 | -1.828 | -5.963 | -1.670 |
| DTE_SST_fast | 43 | -2.385 | 5.669 | 0.865 | -2.377 | -4.130 | -0.640 |

**Gait Cycle Time (GCT)**

**Table S9**. Bayesian Paired Samples T-Test Table

| **Measure 1** | **Measure 2** | **BF_10_** | **Error %** |
| --- | --- | --- | --- |
| DTE_LA_med | DTE_LA_slow | 0.184 | 0.054 |
| DTE_LA_med | DTE_LA_fast | 0355 | 0.041 |
| DTE_RA_med | DTE_RA_slow | 0.329 | 0.042 |
| DTE_RA_med | DTE_RA_fast | 1.075 | 0.022 |
| DTE_SST_med | DTE_SST_slow | 1.955 | 0.015 |
| DTE_SST_med | DTE_SST_fast | 0.333 | 0.042 |

**Table S10**. Descriptive statistics of GCT

|  |  |  |  |  |  | **95% Credible Interval** | |
| --- | --- | --- | --- | --- | --- | --- | --- |
|  | **N** | **Mean** | **SD** | **SE** | **Coefficient of Variation** | **Lower** | **Upper** |
| DTE_LA_med | 43 | -0.903 | 4.675 | 0.713 | -5.180 | -2.341 | 0.536 |
| DTE_LA_slow | 43 | -1.189 | 4.012 | 0.612 | -3.374 | -2.424 | 0.046 |
| DTE_LA_fast | 43 | -0.183 | 2.685 | 0.409 | -14.707 | -1.009 | 0.644 |
| DTE_RA_med | 43 | -0.940 | 4.237 | 0.646 | -4.510 | -2.244 | 0.364 |
| DTE_RA_slow | 43 | -1.463 | 4.825 | 0.736 | -3.298 | -2.948 | 0.022 |
| DTE_RA_fast | 43 | 0.253 | 3.383 | 0.516 | 13.396 | -0.789 | 1.294 |
| DTE_SST_med | 43 | -0.882 | 4.351 | 0.664 | -4.932 | -2.221 | 0.457 |
| DTE_SST_slow | 43 | -2.161 | 4.992 | 0.761 | -2.310 | -3.697 | -0.625 |
| DTE_SST_fast | 43 | -0.138 | 3.591 | 0.548 | -26.077 | -1.243 | 0.967 |

**Single Stance Phase (SSP)**

**Table S11**. Bayesian Paired Samples T-Test

| **Measure 1** | **Measure 2** | **BF_10_** | **Error %** |
| --- | --- | --- | --- |
| DTE_LA_med | DTE_LA_slow | 0.262 | 0.046 |
| DTE_LA_med | DTE_LA_fast | 0.165 | 0.057 |
| DTE_RA_med | DTE_RA_slow | 7.340 | 2.431x10^-7^ |
| DTE_RA_med | DTE_RA_fast | 0.197 | 0.053 |
| DTE_SST_med | DTE_SST_slow | 0.438 | 0.036 |
| DTE_SST_med | DTE_SST_fast | 0.174 | 0.054 |

**Table S12**. Descriptive statistics of SSP

|  |  |  |  |  |  | **95% Credible Interval** | |
| --- | --- | --- | --- | --- | --- | --- | --- |
|  | **N** | **Mean** | **SD** | **SE** | **Coefficient of Variation** | **Lower** | **Upper** |
| DTE_LA_med | 43 | -0.118 | 2.965 | 0.452 | -25.189 | -1.030 | 0.795 |
| DTE_LA_slow | 43 | -0.704 | 3.781 | 0.583 | -5.374 | -1.882 | 0.475 |
| DTE_LA_fast | 43 | -0.105 | 4.001 | 0.610 | -38.142 | -1.336 | 1.126 |
| DTE_RA_med | 43 | -0.530 | 3.136 | 0.478 | -5.920 | -1.495 | 0.435 |
| DTE_RA_slow | 43 | -1.691 | 3.260 | 0.516 | -1.929 | -2.733 | -0.648 |
| DTE_RA_fast | 43 | -0.118 | 4.570 | 0.697 | -38.822 | -1.524 | 1.289 |
| DTE_SST_med | 43 | -0.128 | 3.198 | 0.493 | -25.029 | -1.124 | 0.869 |
| DTE_SST_slow | 43 | -1.084 | 2.749 | 0.424 | -2.536 | -1.941 | -0.228 |
| DTE_SST_fast | 43 | -0.001 | 3.977 | 0.606 | -3918.108 | -1.225 | 1.223 |

**Stance Phase (StP)**

**Table S13**. Bayesian Paired Samples T-Test

| **Measure 1** | **Measure 2** | **BF_10_** | **Error %** |
| --- | --- | --- | --- |
| DTE_LA_med | DTE_LA_slow | 0.378 | 0.039 |
| DTE_LA_med | DTE_LA_fast | 0.172 | 0.056 |
| DTE_RA_med | DTE_RA_slow | 7.041 | 2.586x10^-7^ |
| DTE_RA_med | DTE_RA_fast | 0.176 | 0.055 |
| DTE_SST_med | DTE_SST_slow | 0.520 | 0.033 |
| DTE_SST_med | DTE_SST_fast | 0.168 | 0.055 |

**Table S14**. Descriptive statistics of StP

|  |  |  |  |  |  | **95% Credible Interval** | |
| --- | --- | --- | --- | --- | --- | --- | --- |
|  | **N** | **Mean** | **SD** | **SE** | **Coefficient of Variation** | **Lower** | **Upper** |
| DTE_LA_med | 43 | 0.053 | 1.953 | 0.298 | 36.549 | -0.548 | 0.654 |
| DTE_LA_slow | 43 | 0.548 | 2.385 | 0.368 | 4.353 | -0.195 | 1.291 |
| DTE_LA_fast | 43 | 0.148 | 2.649 | 0.404 | 17.949 | -0.668 | 0.963 |
| DTE_RA_med | 43 | 0.355 | 2.132 | 0.325 | 6.013 | -0.302 | 1.011 |
| DTE_RA_slow | 43 | 1.132 | 2.085 | 0.330 | 1.842 | 0.465 | 1.799 |
| DTE_RA_fast | 43 | 0.190 | 2.994 | 0.457 | 15.739 | -0.731 | 1.112 |
| DTE_SST_med | 43 | 0.087 | 2.028 | 0.313 | 23.410 | -0.545 | 0.719 |
| DTE_SST_slow | 43 | 0.769 | 1.811 | 0.283 | 2.356 | 0.197 | 1.340 |
| DTE_SST_fast | 43 | 0.077 | 2.533 | 0.386 | 33.019 | -0.703 | 0.856 |

**Swing Phase (SwP)**

**Table S15**. Bayesian Paired Samples T-Test

| **Measure 1** | **Measure 2** | **BF_10_** | **Error %** |
| --- | --- | --- | --- |
| DTE_LA_med | DTE_LA_slow | 0.283 | 0.045 |
| DTE_LA_med | DTE_LA_fast | 0.166 | 0.057 |
| DTE_RA_med | DTE_RA_slow | 0.503 | 0.034 |
| DTE_RA_med | DTE_RA_fast | 0.187 | 0.054 |
| DTE_SST_med | DTE_SST_slow | 0.546 | 0.033 |
| DTE_SST_med | DTE_SST_fast | 0.165 | 0.057 |

**Table S16**. Descriptive statistics of SwP

|  |  |  |  |  |  | **95% Credible Interval** | |
| --- | --- | --- | --- | --- | --- | --- | --- |
|  | **N** | **Mean** | **SD** | **SE** | **Coefficient of Variation** | **Lower** | **Upper** |
| DTE_LA_med | 43 | -0.066 | 3.005 | 0.458 | -45.533 | -0.991 | 0.859 |
| DTE_LA_slow | 43 | -0.682 | 3.638 | 0.555 | -5.332 | -1.802 | 0.437 |
| DTE_LA_fast | 43 | -0.120 | 4.038 | 0.616 | -33.784 | -1.362 | 1.123 |
| DTE_RA_med | 43 | -0.469 | 3.218 | 0.491 | -6.865 | -1.459 | 0.522 |
| DTE_RA_slow | 43 | -1.395 | 3.959 | 0.604 | -2.839 | -2.613 | -0.176 |
| DTE_RA_fast | 43 | -0.116 | 4.680 | 0.714 | -40.352 | -1.556 | 1.324 |
| DTE_SST_med | 43 | -0.001 | 3.159 | 0.482 | -2617.778 | -0.973 | 0.971 |
| DTE_SST_slow | 43 | -0.972 | 2.969 | 0.453 | -3.055 | -1.885 | -0.058 |
| DTE_SST_fast | 43 | -0.010 | 4.030 | 0.615 | -392.717 | -1.251 | 1.230 |

**Step Left (LF)**

**Table S17**. Bayesian Paired Samples T-Test

| **Measure 1** | **Measure 2** | **BF_10_** | **Error %** |
| --- | --- | --- | --- |
| DTE_LA_med | DTE_LA_slow | 0.0395 | 0.039 |
| DTE_LA_med | DTE_LA_fast | 3.301 | 7.264x10^-7^ |
| DTE_RA_med | DTE_RA_slow | 0.185 | 0.054 |
| DTE_RA_med | DTE_RA_fast | 0.174 | 0.056 |
| DTE_SST_med | DTE_SST_slow | 0.220 | 0.051 |
| DTE_SST_med | DTE_SST_fast | 1.174 | 0.021 |

**Table S18**. Descriptive statistics of LF

|  |  |  |  |  |  | **95% Credible Interval** | |
| --- | --- | --- | --- | --- | --- | --- | --- |
|  | **N** | **Mean** | **SD** | **SE** | **Coefficient of Variation** | **Lower** | **Upper** |
| DTE_LA_med | 43 | -0.418 | 1.731 | 0.264 | -4.138 | -0.951 | 0.114 |
| DTE_LA_slow | 43 | 0.202 | 2.531 | 0.386 | 12.527 | -0.577 | 0.981 |
| DTE_LA_fast | 43 | 0.351 | 2.323 | 0.354 | 6.621 | -0.364 | 1.066 |
| DTE_RA_med | 43 | 0.095 | 2.625 | 0.400 | 27.612 | -0.713 | 0.903 |
| DTE_RA_slow | 43 | 0.342 | 2.815 | 0.429 | 8.236 | -0.525 | 1.208 |
| DTE_RA_fast | 43 | 0.225 | 2.443 | 0.373 | 10.879 | -0.527 | 0.976 |
| DTE_SST_med | 43 | -0.282 | 2.070 | 0.316 | -7.337 | -0.919 | 0.355 |
| DTE_SST_slow | 43 | 0.060 | 1.920 | 0.293 | 32.151 | -0.531 | 0.651 |
| DTE_SST_fast | 43 | 0.410 | 1.917 | 0.292 | 4.673 | -0.180 | 1.000 |

**Step Right (RF)**

**Table S19**. Bayesian Paired Samples T-Test

| **Measure 1** | **Measure 2** | **BF_10_** | **Error %** |
| --- | --- | --- | --- |
| DTE_LA_med | DTE_LA_slow | 0.0372 | 0.040 |
| DTE_LA_med | DTE_LA_fast | 3.456 | 6.871x10^-7^ |
| DTE_RA_med | DTE_RA_slow | 0.176 | 0.055 |
| DTE_RA_med | DTE_RA_fast | 0.172 | 0.056 |
| DTE_SST_med | DTE_SST_slow | 0.218 | 0.051 |
| DTE_SST_med | DTE_SST_fast | 1.285 | 0.020 |

**Table S20**. Descriptive statistics of RF

|  |  |  |  |  |  | **95% Credible Interval** | |
| --- | --- | --- | --- | --- | --- | --- | --- |
|  | **N** | **Mean** | **SD** | **SE** | **Coefficient of Variation** | **Lower** | **Upper** |
| DTE_LA_med | 43 | 0.428 | 1.754 | 0.267 | 4.096 | -0.112 | 0.968 |
| DTE_LA_slow | 43 | -0.166 | 2.459 | 0.375 | -14.842 | -0.992 | 0.591 |
| DTE_LA_fast | 43 | -0.336 | 2.350 | 0.358 | -6.997 | -1.059 | 0.387 |
| DTE_RA_med | 43 | -0.102 | 2.650 | 0.404 | -25.866 | -0.918 | 0.713 |
| DTE_RA_slow | 43 | -0.285 | 2.774 | 0.423 | -9.748 | -1.138 | 0.569 |
| DTE_RA_fast | 43 | -0.219 | 2.470 | 0.377 | -11.287 | -0.979 | 0.541 |
| DTE_SST_med | 43 | 0.306 | 2.120 | 0.323 | 6.923 | -0.346 | 0.958 |
| DTE_SST_slow | 43 | -0.034 | 1.911 | 0.291 | -55.554 | -0.622 | 0.554 |
| DTE_SST_fast | 43 | -0.407 | 1.917 | 0.292 | -4.709 | -0.997 | 0.183 |

**Two-way Bayesian repeated measure ANOVA**

A two-way (task-by-speed) Bayesian repeated measure ANOVA was performed on all kinematic outcome variables to evaluate possible interactions among cognitive tasks and motor variables. Further post-hoc comparisons were conducted with posterior odds corrected for multiple testing by fixing to 0.5 the prior probability that the null hypothesis holds across all comparisons. Individual comparisons are based on the default t-test with a Cauchy (0, r=1/sqrt(2)) prior.

**Table S21**. Results of Bayesian RM ANOVA on motor parameters considering the factor Task, Speed and Task by Speed.

|  | Cadence | GCL | GCT | SSP | StP | SwP | RF | LF |
| --- | --- | --- | --- | --- | --- | --- | --- | --- |
| Speed | 27.75 * | 1.20x10^6^ * | 11.41 * | 1.69 | 1.57 | 0.48 | 0.25 | 0.26 |
| Task + Speed | 3.19 | 67170* | 0.97 | 0.11 | 0.1 | 0.04 | 0.01 | 0.01 |
| Task | 0.11 | 1.99x10^-4^ | 0.08 | 0.06 | 0.06 | 0.08 | 0.06 | 0.06 |

**Table S22**. Post–Hoc Comparisons of Cadence, Gait Cycle Length and Gait Cycle Time.

|  | Prior Odds | Posterior Odds | BF_10, U_ | Error % |
| --- | --- | --- | --- | --- |
| Cadence |  |  |  |  |
| Slow - Normal | 0.59 | 0.618 | 1.052 | 0.02 |
| Slow - Fast | 0.59 | 712.4 | 1212.8* | 1.37x10^-9^ |
| Normal - Fast | 0.59 | 5.65 | 9.61* | 0.003 |
| GCL |  |  |  |  |
| Slow - Normal | 0.59 | 0.36 | 0.61 | 0.035 |
| Slow - Fast | 0.59 | 0.83 | 1.41 | 0.017 |
| Normal - Fast | 0.59 | 0.09 | 0.15 | 0.12 |
| LA - RA | 0.59 | 0.07 | 0.12 | 0.15 |
| LA - SST | 0.59 | 5.85 | 9.96* | 0.003 |
| RA - SST | 0.59 | 11.14 | 18.97* | 0.002 |
| GCT |  |  |  |  |
| Slow - Normal | 0.59 | 0.06 | 0.1 | 0.17 |
| Slow - Fast | 0.59 | 0.12 | 0.21 | 0.09 |
| Normal - Fast | 0.59 | 0.12 | 0.2 | 0.09 |

1. **ANOVA to control the ORDER OF SESSIONS**

Analysis of variance (ANOVA) on cognitive data (LA – SST) to assess the role of session order in modulating the observed effects. We considered the condition (single task, slower than normal speed, medium speed and faster than normal speed) as a fixed factor, while the order in which participants performed the session (I – II – III – IV day) as a random factor.

**LA**

**Table S23**. Results from ANOVA (Loss-Aversion)

|  | Df | Sum of Squares | Mean Square | F | p-value |
| --- | --- | --- | --- | --- | --- |
| Session | 3 | 0.90 | 0.299 | 0.708 | 0.549 |
| Condition | 3 | 0.68 | 0.225 | 0.53 | 0.662 |
| Session * Condition | 4 | 2.78 | 0.695 | 1.652 | 0.164 |

#### **Figure S2**. Descriptive plots


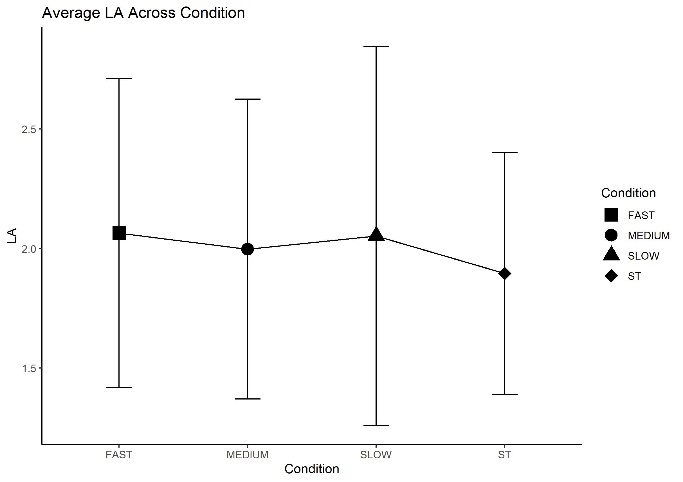

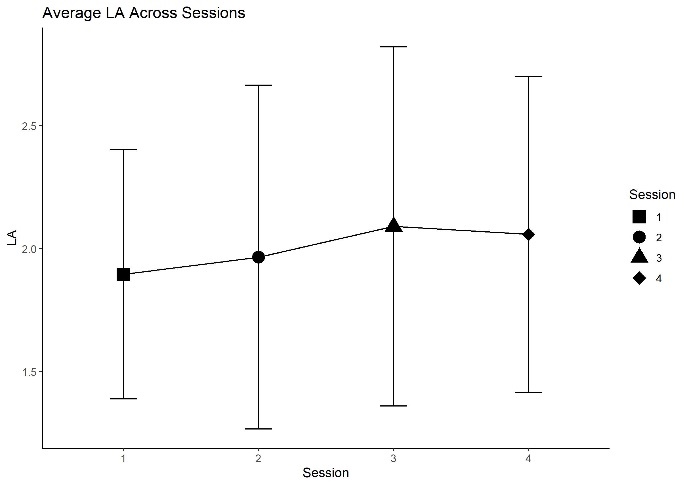


**SST**

**Table S24**. Results from ANOVA (Stop-Signal Task)

|  | Df | Sum of Squares | Mean Square | F | p-value |
| --- | --- | --- | --- | --- | --- |
| Session | 3 | 1984 | 661.5 | 0.486 | 0.693 |
| Condition | 3 | 1599 | 532.9 | 0.391 | 0.76 |
| Session * Condition | 4 | 8485 | 2121.3 | 1.571 | 0.187 |

**Figure S3**. Descriptive plots


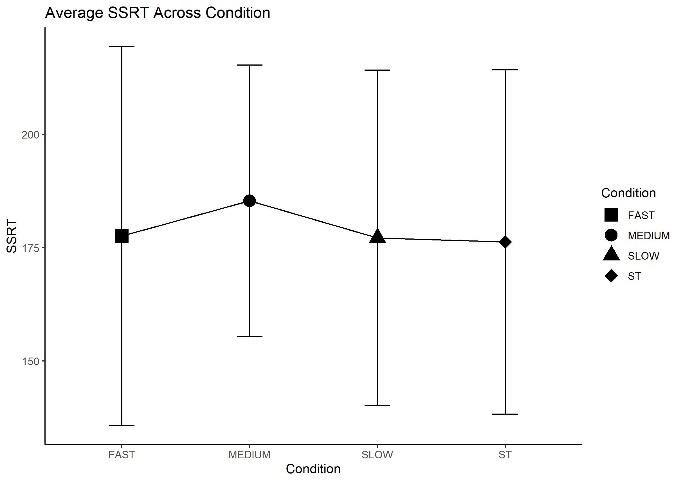

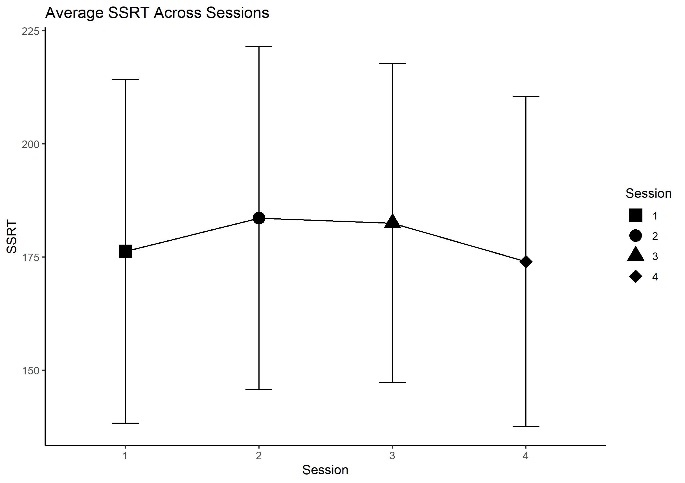


1. **CORRELATION ANALYSIS**

To assess whether a linear relationship could occur between the decrease/increase of the motor performance and the concurrent increase/decrease of cognitive performance a Pearson correlation analysis was computed to measure the direction of this effect for SST and LA.

**Table S25**. Correlation results for DTE at medium speed for SST and DTE at medium speed for motor variables

| Variable | Correlation | P_Value |
| --- | --- | --- |
| Cadence | 0.393 | 0.039 * |
| Stride Length | -0.39 | 0.04 * |
| Gait Cycle Length | -0.13 | 0.51 |
| Gait Cycle Time | -0.411 | 0.03 * |
| Single Stance Phase | 0.045 | 0.821 |
| Stance Phase | -0.046 | 0.816 |
| Swing Phase | 0.055 | 0.781 |
| Step Left | 0.115 | 0.561 |
| Step Right | -0.103 | 0.6 |

**Table S26**. Correlation results for DTE at medium speed for LA and DTE at medium speed for motor variables

| Variable | Correlation | P_Value |
| --- | --- | --- |
| Cadence | -0.05 | 0.783 |
| Stride Length | 0.044 | 0.806 |
| Gait Cycle Length | 0.423 | 0.014 * |
| Gait Cycle Time | 0.054 | 0.765 |
| Single Stance Phase | 0.146 | 0.417 |
| Stance Phase | -0.164 | 0.363 |
| Swing Phase | 0.053 | 0.395 |
| Step Left | 0.096 | 0.596 |
| Step Right | -0.096 | 0.597 |
